# Evaluation of Lateral Ear Canal Ablation (LECA) as a Surgical Treatment Option for External Ear Canal Disease in Lop-Eared Pet Rabbits (*Oryctolagus cuniculus*)

**DOI:** 10.3390/ani15081142

**Published:** 2025-04-16

**Authors:** Anne Willems, Johannes Hetterich, Milena Thöle, Michael Pees, Michael Fehr, Maximilian Reuschel

**Affiliations:** 1 Department of Small Mammal, Reptile and Avian Medicine and Surgery, University of Veterinary Medicine Hannover, Bünteweg 2, 30559 Hannover, Germany; johannes.hetterich@tiho-hannover.de (J.H.); michael.pees@tiho-hannover.de (M.P.); michael.fehr.ir@tiho-hannover.de (M.F.); maximilian.reuschel@tiho-hannover.de (M.R.); 2 Veterinary Clinic Posthausen, Rothlaker Straße 1, 28870 Posthausen, Germany

**Keywords:** rabbit surgery, small mammal, ear disease, lateral ear canal ablation

## Abstract

A soft tissue-filled external ear canal, sometimes also referred to as otitis externa in the veterinary literature, is a prevalent disease in pet rabbits, especially in lop-eared pet rabbits. The authors describe the clinical findings, diagnostic workup, therapy, and outcome of pet rabbits treated surgically for a lateral ear canal ablation (LECA). For the time period between 2015 and 2023, the medical records of two veterinary clinics were evaluated retrospectively. A total of 25 pet rabbits, diagnosed via computed tomography (CT) scans with a soft tissue-filled external ear canal and (if present) middle ear, were identified. All the pet rabbits were evaluated as lop-eared pet rabbits and all were treated surgically using the same surgical technique, LECA. In accordance with Richardson et al., we categorized the different ear filling levels by using the grading system that was established in 2019. The mean follow-up period was 19 days, and in one case complete wound healing was not reached within 131 days after surgery. A total of 7/25 of the pet rabbits were evaluated with wound healing issues. Of the cases, 18/25 did not develop any recurrent ear diseases. Loss to follow-up was observed in 5/25 of the cases and recurrent ear diseases were identified in 2/25 of the pet rabbits. Progression of the ear disease was detected in four cases. CT post-operative images were acquired for 14/25 of the pet rabbits. Based on this study, LECA can be considered a safe surgical method to treat soft tissue-filled external ear canals, especially in lop-eared pet rabbits. Nonetheless, thorough post-surgical management including wound control is critical for therapy success.

## Simple Summary

External ear canal disease is a common disease problem in pet rabbits, especially in lop-eared pet rabbits. The authors describe the clinical findings, diagnostic workup, therapy, and outcome of pet rabbits with defined clinical signs. This is a retrospective clinical case study with data from two different veterinary clinics. Initially, all the pet rabbits underwent computed tomographic imaging (CT) for diagnostic workup. Furthermore, all the pet rabbits were treated surgically, performing a surgical enlargement of the ear canal (lateral ear canal ablation; LECA). A total of 25 pet rabbits underwent surgical procedures between 2015 and 2023 and all of them survived the operation. The mean follow-up period was 19 days. A total of 7/25 pet rabbits were evaluated with wound healing issues. In one case, complete wound healing was not reached within 131 days after surgery. A total of 18/25 pet rabbits did not develop any clinical signs specific to a new ear disease on subsequent examinations. Loss to follow-up was observed in 5/25 of the cases, and recurrent clinical signs were identified in 2/25 of the pet rabbits. In four cases, worsening was observed. CT post-operative images were obtained for 14/25 of the pet rabbits. The surgical method evaluated in this study, LECA, can be considered a safe surgical intervention to treat soft tissue-filled external ear canal diseases in pet rabbits, especially in lop-eared pet rabbits.

## 1. Introduction

Otitis externa is a common reason for the presentation of pet rabbits in veterinary practices [1,2,3]. This disease often develops due to primary dermatitis and the inflammation of the cutaneous tissues of the external ear canal [3]. Generally, lop-eared pet rabbits are predisposed to otitis externa (and otitis media) due to conformational differences in their ear anatomy [4]. Therefore, stenosis of the external ear canal obstructs proper cerumen drainage, resulting in an accumulation of cerumen and erythema [4], establishing an ideal breeding ground for opportunistic microorganisms. A retrospective study evaluating medical issues in 1152 pet rabbits found a prevalence of 3.5% for otitis externa [5]. Another examination reviewing ear conditions in pet rabbits found a prevalence of 25% for ear disease in all lop-eared pet rabbits compared to 10% in not-lop-eared rabbits [6]. Furthermore, the mass of cerumen can independently cause a mechanical obstruction within the ear canal, potentially leading to the development of aural diverticulosis or the eruption of soft tissue material into the middle ear [7,8].

The clinical signs of ear diseases in pet rabbits are considered to be highly variable. In most cases of otitis externa, the affected pet rabbits do not manifest any perceptible clinical signs [9]. The most commonly observed clinical signs associated with otitis externa in pet rabbits include ear shaking, scratching, and torticollis [7]. Even pet rabbits suffering from otitis media showed no clinical signs in 27% of the cases [9]. Clinical signs associated with otitis media/interna manifest primarily in the form of neurological deficits. These include acquired deafness, torticollis, balance disorders, nystagmus, and facial nerve paralysis or spasms [7,10,11]. Definitive diagnosis cannot be achieved through clinical examination alone [10,12,13]. Otoscopy and cytology of the ear canal are simple and quick methods to confirm the diagnosis in dogs and cats [14,15]. Otoscopy, in particular, is a challenging procedure in lop-eared pet rabbits. Due to their anatomical characteristics, the external ear canal is rarely fully visible [7,16]. To clarify the etiology of the soft tissue filling, cytology is recommendable to detect any involvement of inflammation. When combined with computed tomographic imaging, a reliable diagnostic workup technique, it helps to visualize the extent of the disease [13,17]. Richardson et al. introduced a grading system based on computed tomography (CT) evaluation for classification and improved standardized nomenclature of external and middle ear disease in pet rabbits [1].

The surgical technique (LECA) is based on the principle of improving the ventilation of the external ear canal and facilitating the retrograde removal of cerumen with the objective of preventing secondary middle ear disease. The literature has previously discussed LECA for the prevention of otitis media in lop-eared pet rabbits [18]. However, the available data concerning this particular procedure remains limited.

In this study, we describe the clinical findings, diagnostic workup, therapy, and outcome of 25 pet rabbits treated by LECA. We also report the incidence of therapy complications, the bacteria isolated from the pet rabbits’ ears, and their corresponding susceptibility to antimicrobials.

## 2. Materials and Methods

### 2.1. Case Selection

For the period between 2015 and 2023, medical records of two institutions specializing in small mammal medicine and surgery (Department of Small Mammal, Reptile and Avian Medicine and Surgery, University of Veterinary Medicine Hannover, Germany; Veterinary Clinic Posthausen, Germany) were evaluated to identify cases of ear diseases, especially soft tissue-filled external ear canals in pet rabbits. The inclusion criteria were as follows: pet rabbits with a definitive diagnosis through the CT imaging of at least one soft tissue-filled external ear canal, together with subsequent surgical treatment with LECA, having complete and fully comprehensible medical records.

### 2.2. Medical Record Review and CT Scan

The evaluation of the pet rabbits’ medical record data included the rabbits’ sex, age at diagnosis, ear anatomy (lop-eared or not lop-eared), body mass, clinical signs, CT results, comorbidities, surgical method, post-surgical complications, microbiological culture results (aerobic and/or anaerobic), follow-up period, post-surgical treatment, and outcome. CT examination (for University of Veterinary Medicine Hannover using Philips Brilliance 64-Channel, Philips Medical Systems DMC GmbH, Hamburg, Germany until June 2021, followed by Philips IQon Spectral CT, Philips Medical Systems DMC GmbH, and for Veterinary Clinic Posthausen using CT Model Syngo CT VB10 go. Now, Siemens Healthineers AG, Forchheim, Germany) was performed on all the pet rabbits in a transparent plastic box with air holes. The pet rabbits were placed in a sitting position and padded with towels. No sedation or anesthesia was used to perform the procedure. The CT-setting parameters were 80–130 kV and 415–1818 ms. The table feed was 0.6–1.29 mm. No contrast agent was used in any of the cases. Categorization of the soft tissue fillings of the pet rabbits’ ears was based on the results of a study by Richardson et al. [1]: Grade 1—material filling of the external ear canal from the tympanum to the proximal aspect of the acoustic meatus cartilage. No deviation of the tympanum. Grade 2—beyond grade 1 definition, additional lateral pouching of the external ear canal. Grade 3—material filling of the external ear canal from the tympanum to the proximal aspect of the acoustic meatus cartilage. Ventral deviation or obliteration of the tympanum. Grade 4—beyond grade 3 definition, additional lateral pouching of the external ear canal. According to this grading system, a soft tissue-dense filling, even in the absence of cytologic confirmation of inflammatory involvement, is henceforth referred to as otitis.

### 2.3. Surgery and Microbiology Methods

The surgical method performed in both veterinary clinics was, in all cases, a lateral ear canal ablation (LECA), which was first described by Capello [18]. Surgical preparation in accordance with good veterinary practice was carried out in all the cases. This included the appropriate general anesthesia, anesthesia monitoring and maintenance, shaving, and aseptic preparation of the surgical field. The general anesthesia included an intramuscular injection with a combination of ketamine (12–15 mg/kg; Ketamin 10%, cp-Pharma Handelsgesellschaft mbH, Burgdorf, Germany) and medetomidine (0.2 mg/kg; Cepetor^®^ 1 mg/mL, cp-Pharma Handelsgesellschaft mbH). All the pet rabbits were intubated, and anesthesia was maintained with isoflurane (Isofluran CP; cp-Pharma Handelsgesellschaft mbH) in 100% oxygen. During the operation, all the pet rabbits received a continuous intravenous infusion of a complete electrolyte solution (Sterofundin^®^ ISO Infusionslösung, B. Braun Vet Care GmbH, Tuttlingen, Germany) with additional fentanyl (4 µ/kg/h; Fentadon^®^ 50 µg/mL, Dechra Veterinary Products Deutschland GmbH, Aulendorf, Germany) and ketamine (0.3 mg/kg/h; Ketamin 10%, cp-Pharma GmbH, Germany). Following the procedure, all the pet rabbits received subcutaneous antagonization with atipamezole (1 mg/kg; Revertor 5 mg/mL, cp-Pharma Handelgesellschaft mbH). The lateral wall of the ear canal was resected through two parallel incisions following the ear canal from the proximal of the pinna to the ear base. At the base of the ear, the lateral part of the ear canal was dissected so that the opening of the horizontal ear canal became visible. Subsequently, the exposed ear canal epithelium was marsupialized with the cutaneous skin at the edge of the cartilage. Microbiology sampling of the lateral ear canal contents (debris and epithelial lining) was performed during the surgical procedure in all the cases using a sterile transport swab (Amies Agar Gel transsystem, 108C Copan Italia s.p.a., Brescia, Italy) and then transported to a microbiology service (LABOKLIN GmbH & Co. KG, Bad Kissingen, Germany) for aerobic and anaerobic culture, bacterial identification (bacterial culture and MALDI-TOF), and antibiotic susceptibility testing (AST and microdilution method). Post-operatively, the pet rabbits were evaluated for treatment details (analgesic, antibiotic, and supportive care treatment) and the duration of hospitalization.

### 2.4. Complications and Follow-Up

Post-operative complications were defined as clinical conditions that were not pre-existing before the surgical therapy. The recurrence of ear disease of the treated ear, with or without clinical signs, was assessed by anamnestic data provided by the pet owners and clinical follow-ups. Clinical examination and repeated computed tomography scans were evaluated in all the available cases.

## 3. Results

### 3.1. Signalement and Anamnestic Data

In total, 12/25 pet rabbits were female and 13/25 were male. The body mass ranged from 1.65 to 6.39 kg with a median of 2.66 kg. The age range for the pet rabbits at the time of surgery was 2 to 9 years. The mean age was 4.9 years. All the pet rabbits were evaluated as lop-eared pet rabbits (100%). Anamnestically, the pet rabbit owners reported various clinical signs of their animals, such as ear base swelling (7/25), anorexia (3/25), apathy (3/25), stridor (1/25), and facial nerve spasm (1/25). However, the owners did not notice any clinical signs in the majority of cases (17/25). A cytologic examination following a previous otoscopy was not performed in any of the cases.

A total of 21/25 pet rabbits were evaluated with at least one comorbidity at the time of the initial clinical examination. Dental diseases (9/25) were most commonly reported, followed by obesity (5/25), rhinitis (4/25), dacryocystitis (4/25), urinary tract diseases (4/25), and other ophthalmological disorders (3/25). A total of four pet rabbits (4/25) were reported to have no comorbidity at the time of ear disease diagnosis. Obesity was determined using the Body Condition Score (BCS); in rabbits, a score of 5 on a 1 to 5-point scale corresponds to obesity [19].

### 3.2. CT Results Prior to Surgery

By analyzing the CT findings, all grades (I–IV) of soft tissue fillings of the external ear canals were diagnosed. Table 1 details the grades of all the ears before and after surgery for all 25 pet rabbits.

**Figure 1:**
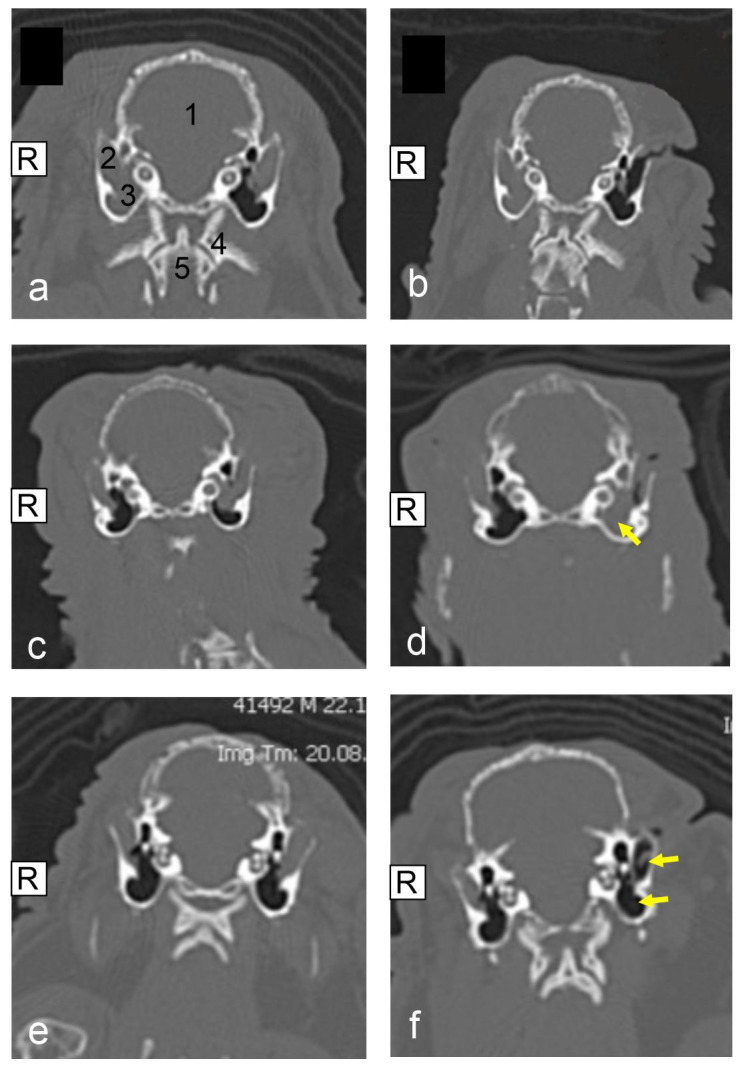
Computed tomographic (CT) image (native, transversal plane, bone window) (**a**–**f**): veterinary clinic Posthausen, Germany); (**a**,**b**): pet rabbit no. 14, (**a**): prior to lateral ear canal ablation (LECA) surgery on the left ear, (1) cranium, (2) external ear canal, (3) middle ear, (4) first cervical vertebra, and (5) second cervical vertebra; (**b**): 365 days after LECA surgery on the left ear, improved ear status, external ear nearly completely filled with air; (**c**,**d**): pet rabbit no. 19, (**c**): prior to LECA surgery on the left ear, (**d**): 240 days after LECA surgery, complete soft tissue filling of the middle ear; (**e**,**f**): pet rabbit no. 20, (**e**): prior to LECA surgery on the left ear, (**f**): 365 days after LECA surgery, improved ear status, external ear nearly completely filled with air.

Relating to a total of 18 pet rabbits with one surgically treated ear, four of those were diagnosed with grade I, and nine pet rabbits with grade II for the external ear canal. In none of these cases was concomitant otitis media identified on the same ear. Two cases had grade III for the external ear canal and both pet rabbits also showed grade I otitis media on the respective ear. A total of three pet rabbits were diagnosed with grade IV for the external ear canal. One of these pet rabbits was also diagnosed with grade I otitis media on the same ear. Both ears were reviewed and categorized for another seven pet rabbits. See Figure 1 for exemplary CT images.

### 3.3. Surgery and Microbiology Findings

In the present study, surgical intervention was performed on the right ear in 11 of the 25 pet rabbits, on the left ear in 7 of the 25 cases, and on both ears (in one surgical procedure) in 7 of the 25 pet rabbits. None of the pet rabbits with external ear disease had a history of otic surgery.

Microbial culture and sensitivity testing were performed in 14/25 pet rabbits. Bacterial growth was detected in 12/25 of the rabbits. The culture findings included *Staphylococcus aureus* (3), *Staphylococcus intermedius* (2), coagulase-negative *Staphylococci* spp. (2), *Corynebacterium* spp. (2), *Peptostreptococcus* sp. (1), *Actinobacillus capsulatus* (1), *Staphylococcus haemolyticus* (1), *Escherischia coli* (1), and methicillin-resistant *Staphylococcus aureus* (MRSA) (1). No bacterial growth was determined in two cases. Furthermore, no microbiological sampling at all was performed in 11 rabbits. A table showing the microbiological results, including resistance characteristics and details of antibiotic treatment, is provided in the Supplementary Materials.

### 3.4. Post-Surgical Therapy

All 25 pet rabbits were administered analgesics prior to and following the procedure of lateral ear canal ablation including meloxicam (0.5 mg/kg PO BID; Meloxicam; Metacam 1.5 mg/mL, Boehringer Ingelheim GmbH, Ingelheim, Germany) and metamizole (50 mg/kg SC BID-TID; Novaminsulfon, 500 mg/mL, Dechra Veterinary Products Deutschland GmbH, Aulendorf, Germany). The pet rabbits were administered buprenorphine (0.03 mg/kg SC BID-TID; Bupresol 0.3 mg/mL, cp pharma Handelsgesellschaft mbH, Germany) at least once post-surgery. If pain signs were observed during the subsequent hospitalization, the administration of buprenorphine was repeated every 6–8 h. The pet rabbits received meloxicam until complete wound healing was achieved. For pain assessment, the facial grimace score from Keating et al. [20] was used, as well as general condition, behavior, and feed intake.

All 25 pet rabbits received antibiotic treatment post-surgery. Upon microbiological results, antibiotic agents were adjusted accordingly if necessary. In Germany, only one commercially available antibiotic (active ingredient enrofloxacin) is licensed for pet rabbits, so this is the first choice for legal reasons. In all the cases, the antibiotic therapy was maintained until the inflammatory reaction resolved and wound healing was completed. If the pet rabbits did not show independent food intake post-surgery, syringe feeding was provided with substitutional herbivore nutrition (20 mL/kg PO TID-QID; Critical Care, Oxbow Animal Health, Oxbow Enterprises, Inc., Omaha, NE, USA). The pet rabbits were hospitalized for one to five days (median 1.8 days) following the surgical procedure.

### 3.5. CT Results Post Surgery

Repeat CT imaging was performed in 14 of the 25 pet rabbits. The time of repeat CT imaging varied between 30 and 762 days post-surgery. One pet rabbit was presented for two follow-up CT examinations. The results of the 14 pet rabbits that received repeat CT imaging are presented in Table 1. In summary, 8 of these 14 pet rabbits showed an improved or partially improved ear grading in the post-operative images based on the status of soft tissue filling. Four of the eight pet rabbits exhibited an improvement in one grade for the external ear canal. Another four pet rabbits showed an improvement of two grades for the external ear canal. Four pet rabbits identified on post-operative CT imaging demonstrated no apparent change to their ear condition. Finally, 2 of the 14 pet rabbits showed a worsening of the soft tissue filling of their ears. In 4 of the 25 cases, the ipsilateral middle ear was included in the disease process. The degree of soft tissue filling of the middle ear remained unchanged in two cases (pet rabbits no. 4 and no. 11). In two cases, the degree of soft tissue filling of the middle ear worsened at the time of the repeated CT images (pet rabbits no. 18 and no. 19). See Figure 1 for exemplary CT images.

### 3.6. Outcome

The mean follow-up period was 19 days, with a range of 12 to 70 days for 24 of the 25 pet rabbits. In one case (pet rabbit no. 24), complete surgical wound healing was not reached at the time of data collection (131 days post-operatively). The rabbit remained in treatment (NSAIDs, local antiseptics, and antibiotics; provided in the Supplementary Materials) for a wound infection with multi-resistant bacteria of the surgical site at day 131 post-operatively. In total, 7 of the 25 pet rabbits were evaluated with post-operative complications, leading to a prolonged follow-up period compared to the 18 pet rabbits without post-operative complications. Complications post-surgery included wound swelling, redness, and profuse mucopurulent discharge and whitish fibrinogenic deposits. One of these seven pet rabbits developed severe crust formation accompanied by localized wound infection, which was confirmed by cytologic examination (pet rabbit no. 7). Additionally, three of those seven pet rabbits showed suture dehiscence issues. Among them, for example, pet rabbit no. 4 was evaluated with an extended follow-up period of 70 days due to post-operative complications including suture dehiscence, wound swelling, and fibrinogenic deposits.

Altogether, 18 of the 25 pet rabbits did not develop any recurrent clinical signs or new ear diseases during the follow-up period. In all 18 cases, the pet owners reported observing an improvement in their rabbits’ activity levels and overall quality of life. In 5 of the 25 cases, it was unknown whether recurrent disorders emerged after initial wound healing was reached due to being lost to follow-up. In 2 of the 25 pet rabbits, recurrent ear diseases were present (543 days and 762 days, respectively, following lateral ear canal ablation), also leading to clinical signs. In pet rabbit no. 1, the owner observed ear-related behavioral changes (increase in ear scratching behavior) 543 days post-surgery. Post-operative images revealed recurrent ear disorders, and the rabbit was subsequently treated conservatively.

The second rabbit with a history of recurrent ear disease (pet rabbit no. 3) revealed macroscopic disorders (whitish secretion, reddened ear canal, and ear base swelling) on the previously operated ear in a routine clinical examination 762 days post-surgery. A subsequent CT scan showed soft tissue filling within the right ear canal, which could be classified as grade I otitis externa. No evidence of middle ear disease was found on the repeated CT images. The pet rabbit was administered analgesics, long-term antibiotics, and topical therapy with an ear cleaner. Subsequently, the rabbit was lost to follow-up.

The post-surgical survival time of the pet rabbits ranged from 204 to 1979 days. Overall, only one rabbit was euthanized due to the consequences of severe ear disease (pet rabbit no. 14). In one of the 25 cases, the progression of otitis externa was visualized via CT scan 224 days after surgery (pet rabbit no. 19). In this case, progression was enhanced due to the further development of otitis media. Prior to bilateral LECA, the pet rabbit exhibited evidence of diverticulosis in the left ear base and a minor focal soft tissue filling of the left middle ear. The post-operative images demonstrated that the left middle ear was fully filled at this point.

## 4. Discussion

Ear disease is a common reason for the presentation of pet rabbits in veterinary practices, and lop-eared pet rabbits seem to be particularly affected [5,6]. Due to the anatomical flexion of the external ear canal in lop-eared pet rabbits [1,19], cerumen can accumulate, potentially resulting in the development of aural diverticulosis or the rupture of the tympanic membrane. As a consequence, the ventilation of the external ear canal may decrease, leading to bacterial overgrowth. This results in chronic ear diseases associated with the pathologic filling of the external ear canal and has a negative impact on the quality of life of pet rabbits [6,21]. In the current scientific literature, the term otitis is predominantly used for isodense soft tissue mass filling of the ears shown in diagnostic imaging (CT scans) [1,10]. This terminology must be reconsidered, as otitis is defined as an inflammation, which should be confirmed by cytologic and/or histologic examination. We have also used this terminology standard as a guide, although further cytologic, pathologic, and histologic studies should be conducted to investigate the exact etiology of this disease complex, particularly in lop-eared pet rabbits.

Based on the evaluation of the survival rate of pet rabbits in this study, the surgical method can be considered a safe treatment. Survival rates depend on management before, during, and after surgery, including anesthesia monitoring, analgesia, temperature surveillance, feeding, and care. In addition, pain management is a key factor in post-operative care. This is evidenced by the fact that rabbits may stop food intake if they are in constant pain and suffer from secondary consequences of the gastrointestinal tract [22,23]. To ensure adequate analgesia, we used a combination of different drug classes, for example, intra-operatively through intravenous infusion in combination with ketamine and fentanyl, and post-operatively through the administration of buprenorphine.

The results of the microbiological examination are consistent with those of a previous study [24]. The aforementioned study documented evidence of Staphylococcus aureus (12.59%) and Corynebacterium latis (9.27%) in pet rabbits with otitis externa [24]. With regard to the versatile studied One Health approach, it is highly recommended that microbiological examinations in connection with ear diseases, especially in cases of otitis in pet rabbits, are performed in a more standardized way. This might facilitate the implementation of individually adapted antibiotic therapy plans [25,26]. In our cases, for example, we used pradofloxacin, trimethoprim/sulfonamide, and marbofloxacin in accordance with this consideration.

A variety of conservative treatments such as topical ear medications (ear cleaners such as Tris-NAC^®^, Nextmune Italy SRL, Palazzo Pignano, Italy, or EpiSqualan^®^, alfavet Tierarzeimittel GmbH, Neumünster, Germany) were used in the medical history of the pet rabbits included in this study. However, all the cases were subsequently treated surgically due to a lack of improvement after conservative treatment. This is in line with the recommendations of previous reviews that discussed surgical methods as a more susceptible therapy for ear disease compared to long-term conservative treatment methods [3,27].

The surgical technique used in pet rabbits in this study is based on the procedure described by Capello in 2006 [18]. That report found a standard healing period of 16–18 days for surgical wounds following lateral ear canal resection and ablation. Another study evaluating the treatment of 48 pet rabbits with lateral ear canal resection and bulla osteotomy including follow-up history found comparable, but slightly longer wound healing times [10]. Also, the latter study identified post-operative complications in 12 of 48 ears (25%). Partial wound dehiscence, para-auricular abscesses, and head tilt, among others, were recorded. A third study on peri-operative complications following partial ear canal ablation and lateral bulla osteotomy identified similar complications within the healing period [12]. Surgical site infection with incision dehiscence and ear base abscesses, among other more non-specific clinical signs, were described.

For the 25 pet rabbits evaluated in our study, the mean post-operative wound healing period of 19 days is in line with the aforementioned studies. Since the surgical site is mainly composed of cartilage tissue known for its poor healing characteristics [27], the authors consider the mean follow-up care period to be appropriate. The post-operative complications, including wound swelling, crusts, fibrinous deposits, and suture dehiscence, found in this study are comparable to the evaluations mentioned above. However, based on the results of this study, in most cases, the post-operative complications did not appear to have a lasting negative effect on the permanent healing outcome. Chronic wound healing issues with a negative outcome were evaluated in only one pet rabbit (4%). Nevertheless, contamination of the surgical field by the infected external ear canal should be expected [28]. For this reason, prophylactic antibiotics and surgical site irrigation with various irrigants are recommended in the literature for dogs and cats [28]. Accordingly, the pet rabbit included in this study received preventive and intra-operative treatment including surgical site irrigation, systemic antibiotics, and the topical administration of either chlorhexidine, sodium chloride solutions, or iodine solution. If persistent inflammation occurs following ear canal resection surgery, continued medical therapy may be appropriate. Due to the risk of peri-operative complications, an adequate medical briefing of the pet rabbit owners should be mandatory prior to any therapy attempt.

The medical therapy regimen for pet rabbit no. 24, which had not achieved complete wound healing within 131 days, consisted of NSAIDs, local antiseptics, and antibiotics. The primary cause of the prolonged wound healing issue is likely to be related to an infection with a multi-resistant strain of *Staphylococcus aureus*. All ear conditions that were not treated surgically or not treated with LECA operation did not meet the inclusion criteria of this study. This might explain the considerably worse states of ear disease in some rabbits. No change or worsening of treatment outcome in some rabbits might be related to the ongoing production of cerumen or continuing problems with its removal. Nevertheless, the aim of LECA should be a physiological, fully ventilated external ear canal. In addition, the focus should be on improving quality of life and, if clinical signs were previously present, on resolving these clinical conditions. In terms of the subjective perceptions of the attending veterinarians and the feedback received from the rabbits’ owners, an enhancement in quality of life and an increase in activity level were observed. Unfortunately, these assessments could not be verified objectively. Further investigations are needed to gain a more comprehensive understanding of the surgery technique and its mid-term and long-term effects on the pet rabbits’ welfare.

In 4 of the 25 cases, the LECA surgical technique was selected despite the presence of middle ear involvement. The rationale behind this approach was to facilitate more effective ear cleaning and flushing with the aim of preventing the progression of middle ear disease. Two of those four cases demonstrated a stable condition, while the other two exhibited a worsening of middle ear involvement. The results of this study indicate that the prognosis is less favorable in cases where the middle ear was previously involved in the disease process. Further studies with a larger sample size are necessary to gain a deeper understanding of this condition. For one rabbit suffering from severe bilateral ear disease and other comorbidities (pet rabbit no. 4), the surgical therapy method may have been inappropriate. Especially in the presence of otitis media, a technique for treating the outer ear should be combined with bulla osteotomy [3,10,29]. In this particular case, however, the owners of the rabbit decided against bulla osteotomy.

Alternatively, it has been described that in mild otitis externa, especially in not-lop-eared pet rabbits, medical therapy using flushing solutions and adequate antibiotics can be effective [3]. However, one study already described in 2017 that lop-eared pet rabbits with severe bacterial infections do not respond well to medicinal treatment alone [2]. More specifically, treatment with medication alone is often challenging and not worthwhile [3]. The authors also subjectively support the assessments raised in that study. Currently, there is a lack of studies evaluating and comparing the surgical and conservative treatment options and their respective outcomes for otitis externa disease in pet rabbits.

As an alternative surgical technique to LECA, lateral ear canal stoma surgery should be mentioned [7,18]. This procedure allows for a more effective continuation of therapy with flushing and local therapeutics. Manchinelli characterizes this approach as a partial lateral resection [3]. Nevertheless, experience has demonstrated that this approach is often not a long-term solution. Other surgical techniques that have been performed on rabbit ears include a total ear canal ablation (TECA) and a partial ear canal ablation (PECA) [3,18,29]. Total ear canal ablation is recommended in the case of neoplasia or stenosis. It can be performed solely or in combination with a lateral bulla osteotomy (LBO). None of our cases suffered from neoplasia, for which a TECA/TECALBO would have been necessary. PECA can also be performed alone or in combination with LBO. Further large-scale studies are required to provide clarification regarding recommended surgical techniques for the treatment of otitis in pet rabbits.

Limitations: The retrospective character of the study did not allow active measures to improve data acquisition. Furthermore, the limited number of pet rabbits included in our study and the variable clinical and microbiological status limited the final assessment of the association between lateral ear canal ablation and the benefits for the clinical outcome. Therefore, we decided not to draw statistical conclusions but to refer to further studies on more defined clinical cases.

## 5. Conclusions

Lateral ear canal ablation (LECA) in pet rabbits suffering from bacterial otitis externa or accumulated cerumen masses seems to be a safe surgical method according to the results of this study. A prolonged healing period with post-operative complications such as surgical site infections are treatment complications that must be considered. This is the first study assessing LECA including the medium- to long-term outcomes. Further studies involving a higher number of pet rabbits and other research variables like pathologic and histologic examinations are needed to collect advanced data about this pivotal disease in pet rabbits.

## Figures and Tables

**Table 1:** Comparison of otitis grades based on computed tomographic (CT) images for both ears before and after lateral ear canal ablation in 25 pet rabbits. Fields marked with n.t. (not tested) did not receive control CT scans.

Pet Rabbit No.	Surgical Ear Side	Right External Ear Prior to Surgery	Right External Ear After Surgery	Right Middle Ear Prior to Surgery	Right Middle Ear After Surgery	Left External Ear Prior to Surgery	Left External Ear After Surgery	Left Middle Ear Prior to Surgery	Left Middle Ear After Surgery	Time of CT Control in Days After Surgery
1	left	1	1	0	0	4	3	0	0	543
2	right	1	n.t.	0	n.t.	3	n.t.	1	n.t.	n.t.
3	right	2	2	0	0	1	1	0	0	762
4	left	3	2	2	0	4	3	3	3	66
5	right	2	n.t.	0	n.t.	0	n.t.	0	n.t.	n.t.
6	right	2	n.t.	0	n.t.	2	n.t.	0	n.t.	n.t.
7	right	2	n.t.	0	n.t.	2	n.t.	0	n.t.	n.t.
8	right	2	3	0	0	1	1	0	0	244
9	left	1	2	0	0	2	2	0	0	134
10	both	3	n.t.	1	n.t.	2	n.t.	0	n.t.	n.t.
11	left	2	1	0	0	3	1	1	1	364
12	right	4	3	0	0	1	1	0	0	368
13	right	2	n.t.	0	n.t.	3	n.t.	1	n.t.	n.t.
14	left	3	3	3	3	2	0	0	0	365
15	both	1	n.t.	0	n.t.	1	n.t.	1	n.t.	n.t.
16	right	1	n.t.	0	n.t.	0	n.t.	0	n.t.	n.t.
17	both	1	n.t.	0	n.t.	1	n.t.	0	n.t.	n.t.
18	right	1	3	0	2	3	0	3	0	90
19	left	1	1	0	0	3	1	1	2	224
20	left	1	1	0	0	2	0	0	0	365
21	both	1	n.t.	0	n.t.	3	n.t.	1	n.t.	n.t.
22	right	1	1	0	0	3	0	1	0	180
23	both	1	0	0	0	1	0	0	0	90
24	both	1	1	0	0	1	0	0	0	30
25	both	1	n.t.	0	n.t.	1	n.t.	0	n.t.	n.t.

## Data Availability

The data presented in this study are openly available in a repository at: https://doi.org/10.5281/zenodo.14925044.

## Supplementary Materials

The following supporting information can be downloaded at https://www.mdpi.com/article/10.3390/ani15081142/s1, Table S1: Microbiological results and antibiotic treatment.
