# Supplementary material for: Evaluation of Lateral Ear Canal Ablation (LECA) as a Surgical Treatment Option for External Ear Canal Disease in Lop-Eared Pet Rabbits (Oryctolagus cuniculus)
Source: Animals (Basel). 2025 Apr 16;15(8):1142. doi: 10.3390/ani15081142 (PMC12024415; doi:10.3390/ani15081142)
Supplement: Supplementary file 1 [file animals-15-01142-s001.zip › animals-3524464-supplementary.pdf]

Patient animal	Detected bacteria	Result of antibiogram	Treatment
**1**	coagulase-negative staphylococci	enrofloxacin intermediate, susceptible to all the others	chloramphenicol 35mg/kg BID for 36 d and iodine locally
**2**	*S. intermedius*, *Pasteurella* sp.	no antibiogram was compiled	enrofloxacin 7mg/kg BID for 10 d
**3**	*Corynebacterium* spec.	susceptible to all	enrofloxacin 7mg/kg BID for 14 d
**4**	*S. aureus*	susceptible to all	chloramphenicol 35mg/kg BID for 30 d and iodine locally
**5**	*Peptostreptococcus spec.*, coagulosenegative staphylococci	no antibiogram was compiled	enrofloxacin 7mg/kg BID for 14 d
**6**	Not tested.	.	chloramphenicol 35mg/kg BID for 16 d
**7**	*S. aureus*, *Corynebacterium* sp.	resistant to ampicillin and penicillin G, erythromycin intermediate, susceptible to all the others	enrofloxacin 7mg/kg BID and metronidazol 20mg/kg BID both for 24 d and iodine locally
**8**	*Actinobacillus capsulatus*, *S. intermedius*	resistant to clindamycin susceptible to all the others	enrofloxacin 7mg/kg BID and metronidazol 20mg/kg BID both for 27 d and iodine locally
**9**	*S. aureus*	resistant to polymyxin, susceptible to all the others	enrofloxacin 7mg/kg BID and metronidazol 20mg/kg BID both for 17 d
**10**	negative	.	marbofloxacin 4mg/kg SID for 16 d
**11**	negative	.	Pradofloxacin 5mg/kg SID over 14 d
**12**	*S. haemolyticus*	resistant to ceftazidim, spiramycin, tylosin, tilmicosin, susceptible to all the others	pradofloxacin 5mg/kg SID for 14 d
**13**	Not tested	.	trimethoprim 6mg/kg plus sulfonamid 30mg/kg BID for 14 d
**14**	*E. coli*	resistant tomacrolides, lincosamides, penicillin G, amoxicillin, ampicillin, cephalexin intermediate, susceptible to all the others	marbocyl 4mg/kg SID for 15 d and iodine locally
**15**	Not tested	.	enrofloxacin 10mg/kg SID for 14 d and iodine locally
**16**	*Cutibacterium acnes*	resistant to metronidazol, susceptible to all the others	trimethoprim 6mg/kg plus sulfonamid 30mg/kg BID for 20 d and iodine locally
**17**	Not tested	.	marbocyl 4mg/kg SID for 15 d and iodine locally

**18**	Not tested	.	marbocyl 4mg/kg SID for 14 d and iodine locally
**19**	Not tested	.	enrofloxacin 10mg/kg SID for 14 d and iodine locally
**20**	Not tested	.	enrofloxacin 10mg/kg SID for 14 d and iodine locally
**21**	Not tested	.	enrofloxacin 10mg/kg SID for 10 d and iodine locally
**22**	Not tested	.	enrofloxacin 10mg/kg SID for 15 d and iodine locally
**23**	Not tested	.	enrofloxacin 10mg/kg SID for 14 d and iodine locally
**24**	*Staphylococcus aureus (Methicillin* resistant*)*	resistent to all penicilline, fluoroquinolones and cephalosporins, all other sensitive	enrofloxacin 10mg/kg SID for 20 d and iodine locally; after that marbofloxacin 4mg/kg SID and metronidazol 20mg/kg BID for 7 days; after that doxycyclin 5mg/kg BID for 21 days; after that trimethoprim 6mg/kg plus sulfonamid 30mg/kg BID for 28 days; after re-operation oxytetracyclin (terramycin) 10 mg/kg every 48 h for 25 days
**25**	Not tested	.	enrofloxacin 10mg/kg SID for 14 d and iodine locally
